# Treatment of perimenopausal depressive disorder with acupuncture combined with traditional Chinese medicine decoction: A systematic review and meta-analysis

**DOI:** 10.1097/MD.0000000000048396

**Published:** 2026-04-24

**Authors:** Wenya Huang, Le Zhang, Zhonghao Fang, Yue Dong, Zi Wang, Zhan Gao, Kuok Tong Lei

**Affiliations:** a Southern Medical University Hospital of Integrated Traditional Chinese and Western Medicine, Southern Medical University, Guangzhou, China; b Institute of TCM-Related Comorbid Depression, School of Integrative Medicine, Nanjing University of Chinese Medicine, Nanjing, China; c Department of Biochemistry and Molecular Biology, College of Pharmacy, Hebei University of Chinese Medicine, China.

**Keywords:** acupuncture, depression, meta-analysis, perimenopausal period, traditional Chinese medicine decoction

## Abstract

**Background:**

In recent years, an increasing number of randomized controlled trials (RCTs) have investigated acupuncture combined with traditional Chinese medicine (TCM) decoction for the treatment of perimenopausal depressive disorder (PDD). However, high-quality evidence regarding its efficacy and safety remains limited. This study aimed to systematically evaluate the effectiveness and safety of this combined therapy and to identify optimal treatment strategies for PDD.

**Methods:**

RCTs evaluating acupuncture combined with TCM decoction for PDD, published up to April 2021, were systematically searched in China National Knowledge Infrastructure, VIP, Wanfang, PubMed, Web of Science, the Cochrane Library, Sinomed, and Embase. Data were analyzed using Review Manager 5.4 in accordance with the Cochrane Handbook for Systematic Reviews of Interventions (Version 5.1).

**Results:**

A total of 9 studies involving 601 participants were included. Compared with TCM decoction alone, the combined therapy significantly improved the clinical effective rate (RR = 1.18, 95% confidence interval [CI]: 1.07–1.29), reduced HAMD scores (standardized mean difference [SMD] = −0.87, 95% CI: −1.08–−0.65), Kupperman scores (SMD = −0.61, 95% CI: −1.14–−0.08), and self-rating depression scale scores (mean difference [MD] = −13.58, 95% CI: −18.67–−8.49), and increased estradiol (17β-estradiol) levels (MD = 7.90, 95% CI: 2.90–12.91). Compared with Western medicine, the combined therapy also showed higher clinical effectiveness (RR = 1.16, 95% CI: 1.02–1.33), lower HAMD scores (SMD = −0.54, 95% CI: −0.90–−0.17), and higher estradiol (17β-estradiol) levels (MD = 17.47, 95% CI: 10.12–24.82). The incidence of adverse reactions was lower in the combined therapy group (0.3%) than in the control group (3.3%).

**Conclusion:**

Acupuncture combined with TCM decoction appears to be an effective and safe treatment for PDD, showing superior outcomes compared with TCM decoction alone or Western medicine. However, further large-scale, high-quality RCTs are needed to confirm these findings.

## 1. Introduction

Perimenopausal depression (PDD) is a clinically significant syndrome characterized by depressive episodes during the menopausal transition, a period encompassing the years immediately before and after menopause.^[1]^ The decline in estrogen levels is a key contributor, leading to persistent emotional disturbances, including depression, anxiety, and autonomic dysfunction.^[2–6]^ Women in the perimenopausal phase are at a substantially elevated risk for depression, with prevalence rates reported as high as 36% globally.^[7]^ The prevalence in China is documented at 25.9%^[8]^ and is steadily increasing.^[9]^This condition not only severely impacts quality of life but also poses a significant public health burden due to its association with high rates of recurrence and suicidality.^[10]^

**Figure 1.**
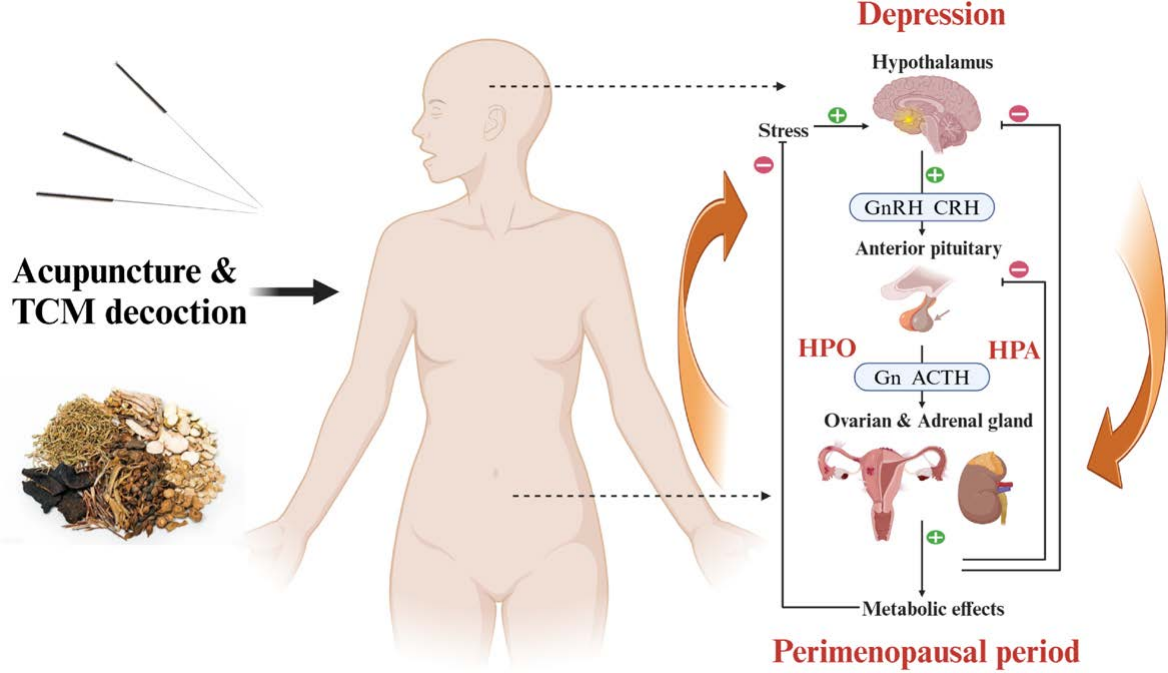
**Graphical abstract: Mechanism of Acupuncture and TCM decoction in treating perimenopausal depressive disorder.** The pathogenesis of perimenopausal depression is primarily due to various hormone dysfunctions and the decline in ovarian function before and after menopause. Positive feedback (+) increases the synthesis or secretion of the GnRH and pituitary gonadotropin. On the other hand, the synthesis or secretion of GnRH and pituitary gonadotropin is called negative feedback (-). Reduced ovarian function and estrogen levels lead to negative feedback regulation of the hypothalamus-pituitary-ovarian axis (HPO) and the hypothalamic-pituitary-adrenal axis (HPA).The negative feedback regulation of HPO and HPA results in alterations across multiple hormone dysfunctions, including the change of gonadotropin-releasing hormone (GnRH), corticotropin-releasing hormone (CRH), gonadotropins (Gn), and adrenocorticotropic hormone (ACTH), causing metabolic effects and producing stress on the hypothalamus, ultimately leading to depression. TCM = traditional Chinese medicine.

The pathogenesis of PDD is complex and multifactorial (see Fig. 1), involving dysregulation of the hypothalamic-pituitary-ovarian axis and hypothalamic-pituitary-adrenal axis (HPA), alongside impairments in monoaminergic neurotransmitter systems (e.g., serotonin, norepinephrine).^[11–13]^ Current mainstream treatments primarily include hormone replacement therapy (HRT), antidepressants, or their combination.^[14]^ However, these options are often limited by side effects, dependency risks, and suboptimal patient compliance, underscoring the need for complementary therapeutic strategies.^[15]^

Traditional Chinese medicine (TCM), including acupuncture and herbal decoctions, has shown promise in managing perimenopausal symptoms.^[4,16]^ While acupuncture is established as a non-pharmacological intervention for depression, and Chinese herbal medicine offers systemic regulation, their combined use represents an integrative approach hypothesized to yield synergistic, multi-target benefits. However, evidence for this specific combination remains fragmented and inadequately synthesized.

Critically, while several systematic reviews have evaluated acupuncture or Chinese herbal medicine individually for menopausal depression, there is a distinct lack of high-qualitymeta-analyses focusing specifically on their combined efficacy for PDD. Existing reviews often possess a broader scope (e.g., all menopausal symptoms) or aggregate various non-pharmacological therapies, thereby failing to isolate and critically assess this particular integrative regimen against relevant active controls. Therefore, this meta-analysis aims to fill this identified niche by providing a focused and updated synthesis of randomized controlled trial (RCT) evidence. It specifically evaluates the efficacy and safety of acupuncture combined with TCM decoction versus active controls (TCM decoction alone or Western medicine) for the treatment of PDD, thereby seeking to establish a clearer evidence base for this combined therapeutic strategy.

This meta-analysis aims to evaluate the short-term clinical efficacy and safety of acupuncture combined with Chinese herbal medicine decoction in treating PDD. Focusing on adult perimenopausal women diagnosed with depressive disorder, the study prioritizes core outcome measures including clinical effectiveness rate, standardized depression scale scores (e.g., HAMD), key hormonal levels (estradiol), and the incidence of adverse events. The objective is to provide an evidence-based foundation for future scientific research and clinical practice. We hypothesize that the combined therapy of acupuncture and Chinese herbal medicine decoction will be superior to monotherapies (Chinese herbal medicine decoction alone or Western medicine) in improving depressive symptoms and hormonal levels, while exhibiting a more favorable safety profile.

## 2. Methods

The systematic review with meta-analysis adhered to the Preferred Reporting Items for Systematic Reviews and Analyses guidelines. Our protocol was conducted following the Preferred Reporting Items for Systematic Reviews and Analyses schema and was registered at the International Platform of Registered Systematic Review and Meta-analysis Protocols under registration number International Platform of Registered Systematic Review and Meta-analysis Protocols 202210122. This study is a systematic review and meta-analysis based on previously published studies; therefore, ethical approval and informed consent were not required. The detailed search strategies for all databases are provided in the Supplementary Methods, Supplemental Digital Content, https://links.lww.com/MD/R716.

### 2.1. Article searching

**Figure 2.**
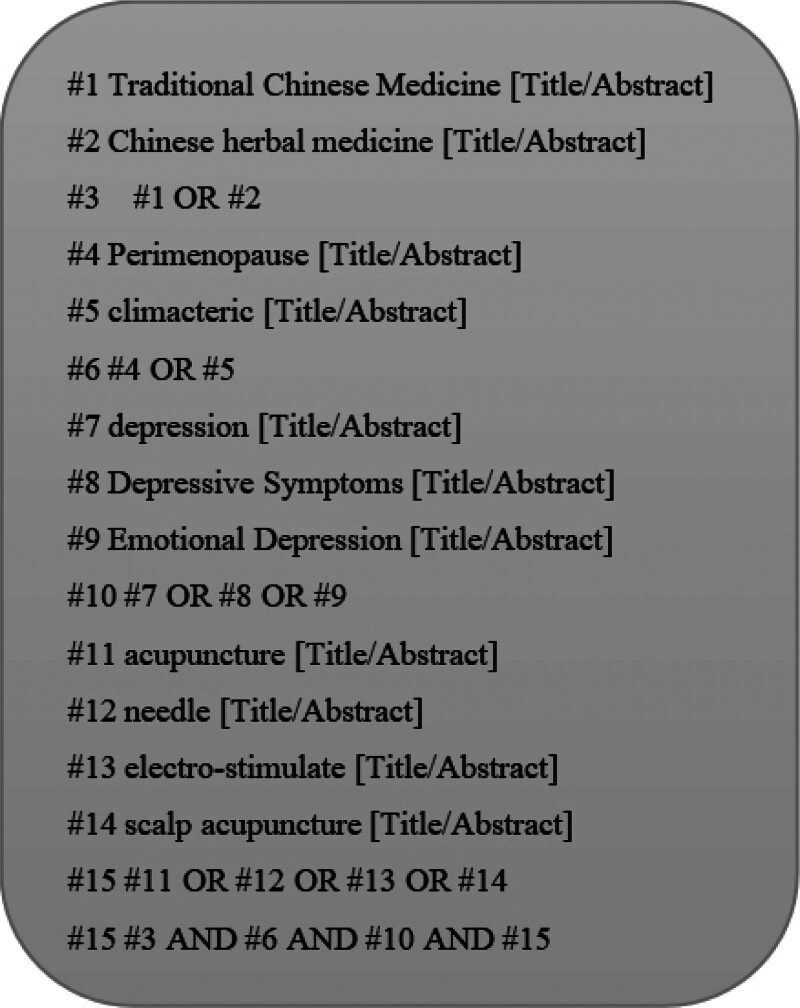
**Flowchart of the literature screening process**. This flowchart illustrates the systematic approach used to screen literature for inclusion in the study. The process starts by identifying key terms such as “Traditional Chinese Medicine,” “Chinese herbal medicine,” and “perimenopause,” which are used to search the literature database. The flowchart proceeds with additional search criteria, including terms related to depression, acupuncture, and other therapeutic methods. Logical combinations (OR/AND) of terms are applied to refine the selection, ensuring that only relevant articles are included in the final dataset.

We searched 5 major Chinese databases China National Knowledge Infrastructure, Wanfang, VIP, and Sinomed) and 4 international databases (PubMed, Web of Science, Embase, and The Cochrane Library) for studies on acupuncture combined with TCM decoction for treating PDD. Keywords included “climacteric,” “perimenopause,” “depression,” “depressive symptoms,” “emotional depression,” “Chinese herbal medicine,” “TCM,” “acupuncture,” “electro-stimulation,” “scalp acupuncture,” and “needle.” The search covered the period from the inception of each database to April 2021. Detailed search strategies for each database are provided below. The search strategy used for PubMed is provided (Fig. 2). The remaining database strategies are shown in the Supplementary Material, Supplemental Digital Content, https://links.lww.com/MD/R716.

### 2.2. Inclusion criteria

For inclusion, we now specify: “Study design: parallel-group RCTs; Participants: perimenopausal women diagnosed with depressive disorder according to established criteria (e.g., DSM-IV/V, Chinese classification of mental disorders, 3rd Edition, or HAMD score thresholds as specified in primary studies); Intervention: Acupuncture combined with TCM decoction.”

#### 2.2.1. Research type

RCT, whether blinded or not, was conducted

#### 2.2.2. Research object

The patient is diagnosed with perimenopausal depression, regardless of age or race. The diagnostic criteria for depression conform to the third edition of the Chinese classification of mental disorders, 3rd Edition, the Diagnostic and Statistical Manual of Mental Disorders, and the Perimenopausal diagnosis “Diagnostic Criteria for Gynecological Diseases.”

#### 2.2.3. Intervention measures

The experimental group was treated with a combination of acupuncture and Chinese herbal medicine, while the control group was treated with either antidepressant drugs or Chinese herbal medicine.

#### 2.2.4. Outcome indicators

Clinical effective rate; HAMD score (Hamilton Depression Scale); Kupperman index; estradiol content; SDS score.

### 2.3. Exclusion criteria

Unclear diagnosis of depression or other diseases: “Studies where the diagnosis of depression was ambiguously reported (e.g., self-reported symptoms without clinical assessment) or where participants had comorbid major psychiatric (e.g., bipolar disorder, schizophrenia) or severe somatic diseases that could independently confound depression or treatment response.” This clarification helps distinguish PDD from depression secondary to other clear pathologies.

### 2.4. Data extraction and risk of bias assessment

Two researchers independently conducted the literature selection and data extraction process based on the predefined inclusion and exclusion criteria. Any disagreements encountered were resolved through discussion between the 2 researchers or, when necessary, by consulting a third researcher. The selection process involved an initial screening of titles and abstracts, followed by a full-text review of the potentially eligible studies to finalize the included literature. The finalized list was cross-checked to ensure consistency.

For each included study, data were extracted using a standardized form. Beyond general study characteristics and outcomes, particular attention was paid to extracting detailed methodological information critical for assessing the risk of bias. This included specifics on random sequence generation (e.g., use of a random number table, computer-generated randomization), allocation concealment (e.g., sealed envelopes, central allocation), the implementation of blinding (of participants, personnel, and outcome assessors), handling of incomplete outcome data, and reporting of dropouts and intention-to-treat analysis. These extracted details formed the basis for the subsequent systematic assessment of the risk of bias for each individual study.

### 2.5. Quality evaluation

The 6 items of the risk of bias assessment tool recommended by the Cochrane Handbook 5.2.3 were used to evaluate the included studies, and the RevMan 5.4 software was used to generate a risk of bias map. The content includes the generation of random sequences, allocation concealment, implementation of blinding methods (blinding of subjects and implementers, and blinding of research outcomes), data integrity, selective publication, and other biases. All these biases can be classified into 3 levels: “low risk,” “unclear,” and “high risk.” Cross-check the above results and discuss and resolve any differences if they exist.

Potential sources of clinical and methodological heterogeneity were prespecified and included: type of control group (TCM decoction vs Western medicine), intervention characteristics (acupuncture/TCM formula details), treatment duration, and baseline severity. These were planned to be explored via subgroup analysis or meta-regression if sufficient studies were available. Statistical heterogeneity was assessed using the *I*^2^ statistic.

### 2.6. Statistical analysis

RevMan 5.4 software was used to analyze the data and conduct a Meta-analysis. For dichotomous data, odds ratio, risk ratio (RR), and 95% confidence interval (CI) were used as the statistical effect size. For Continuous data, use the mean difference (MD) or standardized mean difference (SMD) along with the 95% CI as the statistical effect size.

### 2.7. Ethics

The data we used are based on previously published research, all of which has been ethically approved. Therefore, additional ethical approval is not required.

## 3. Results and analysis

### 3.1. Literature search results

**Figure 3.**
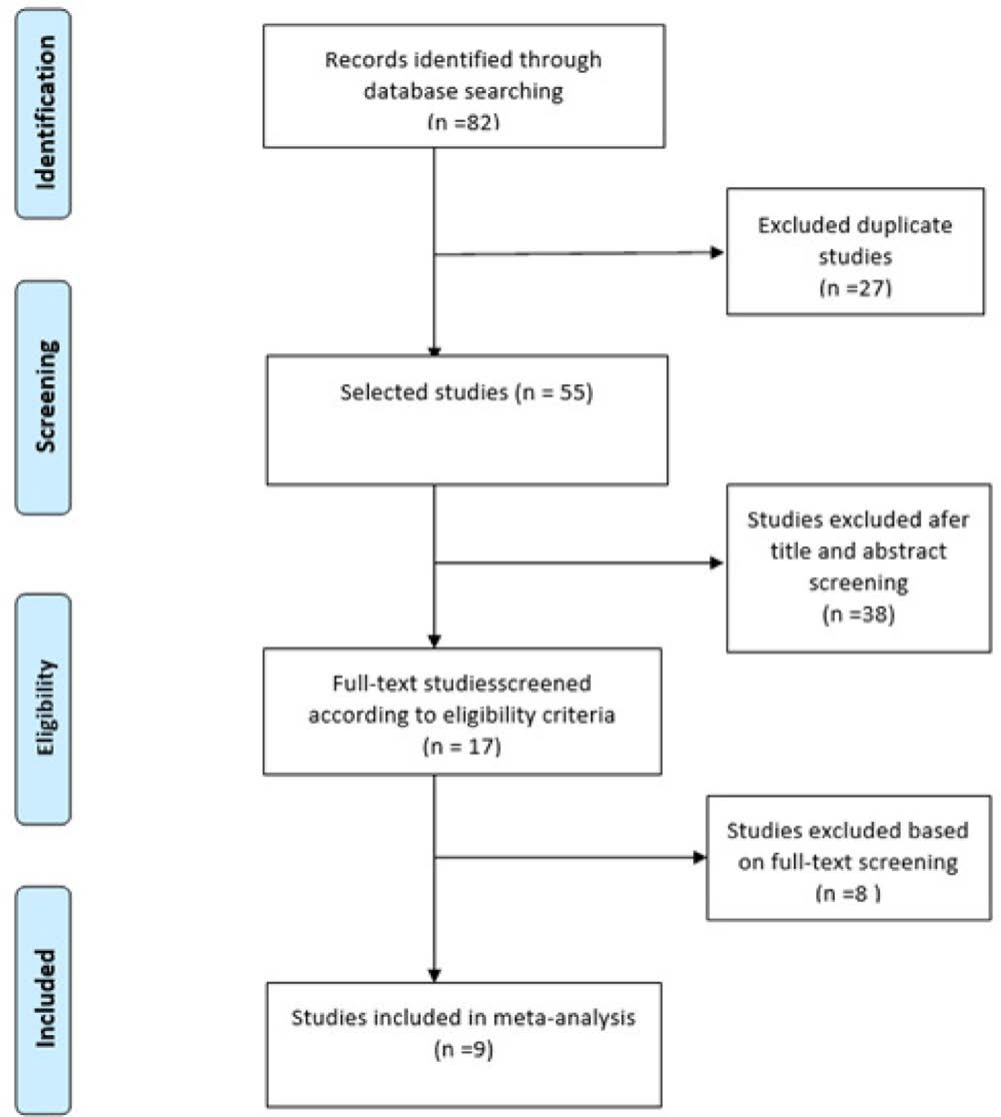
**Document retrieval flow chart and results**. This flow chart illustrates the process and results of the document retrieval and screening for inclusion in a meta-analysis. Initially, 82 records were identified through database searching. After removing duplicate studies, 55 studies were selected for further screening. Following title and abstract screening, 38 studies were excluded, leaving 17 studies for full-text screening based on eligibility criteria. After full-text review, 8 additional studies were excluded, resulting in 9 studies being included in the final meta-analysis.

Using a computer to search the database, we found 33 articles on China National Knowledge Infrastructure, 31 articles on Wanfang, 7 articles on VIP, 2 articles on PubMed, 5 articles in the Cochrane Library, and 4 articles on Web of Science, with no articles found by other means. In total, 82 articles were retrieved, of which 9 were finally included (Figs 2 and 3).

### 3.2. Table of baseline characteristics of the included studies

A total of 9 RCTs were included, with a total of 601 patients: 301 in the treatment group and 300 in the control group. Two control groups received antidepressant drugs, and the rest received Chinese herbal medicine (Table 1).

**Table 1:** Basic characteristics of the included studies.

Included in the study	Group	Sample	Average age	Intervention	Course of treatment	Outcome indicators
Guozhen Chen 2010 ^[17]^	Therapy group	30	49.5	Acupuncture + control	8 wk	①②④
	Control group	30	48.1	Nourishing Kidney, Soothing Liver, and Ningxin Recipe		
Xiaolan Shi 2011 ^[18]^	Therapy group	40	52.2	Acupuncture + control	1 mo	①②③
	Control group	40	52.3	Chinese medicine decoction		
Xiaolan Shi 2012 ^[19]^	Therapy group	30	53.03	Acupuncture + control	1 m	④
	Control group	30	52.23	Chinese medicine decoction		
Yuqing Xie 2013 ^[20]^	Therapy group	30	53	Acupuncture + Xiaoyao San	1 mo	①②
	Control group	30	51.5	Antidepressant drugs		
Huiling Zhu 2013 ^[21]^	Therapy group	30	50.21	Acupuncture + Zishen Shugan decoction	2 mo	①②
	Control group	30	49.73	Antidepressant drugs		
Hongli Huang 2016 ^[22]^	Therapy group	30	Unclear	Acupuncture + control	12 wk	①②③④
	Control group	30	Unclear	Tonifying the kidney, relieving depression, and clearing the heart recipe		
Meijuan Lv 2019 ^[23]^	Therapy group	50	47.5	Acupuncture + control	12 wk	①②③
	Control group	50	49.6	Tonifying the kidney, relieving depression, and clearing the heart recipe		
Xiaolin Yang 2019 ^[24]^	Therapy group	31	49.71	Acupuncture + control	8 wk	⑤
	Control group	32	51.56	Tonifying the kidney and soothing liver recipe		
Gu Ting 2020 ^[25]^	Therapy group	30	49	Acupuncture + control	12 wk	①②③⑤
	Control group	28	50	Happy san		

① Total clinical effective rate; ② Hamilton depression scale (HAMD); ③ Kupperman index; ④ Estradiol; ⑤ Self-rating depression scale (SDS).

### 3.3. Methodological quality evaluation form of the included studies

**Figure 4.**
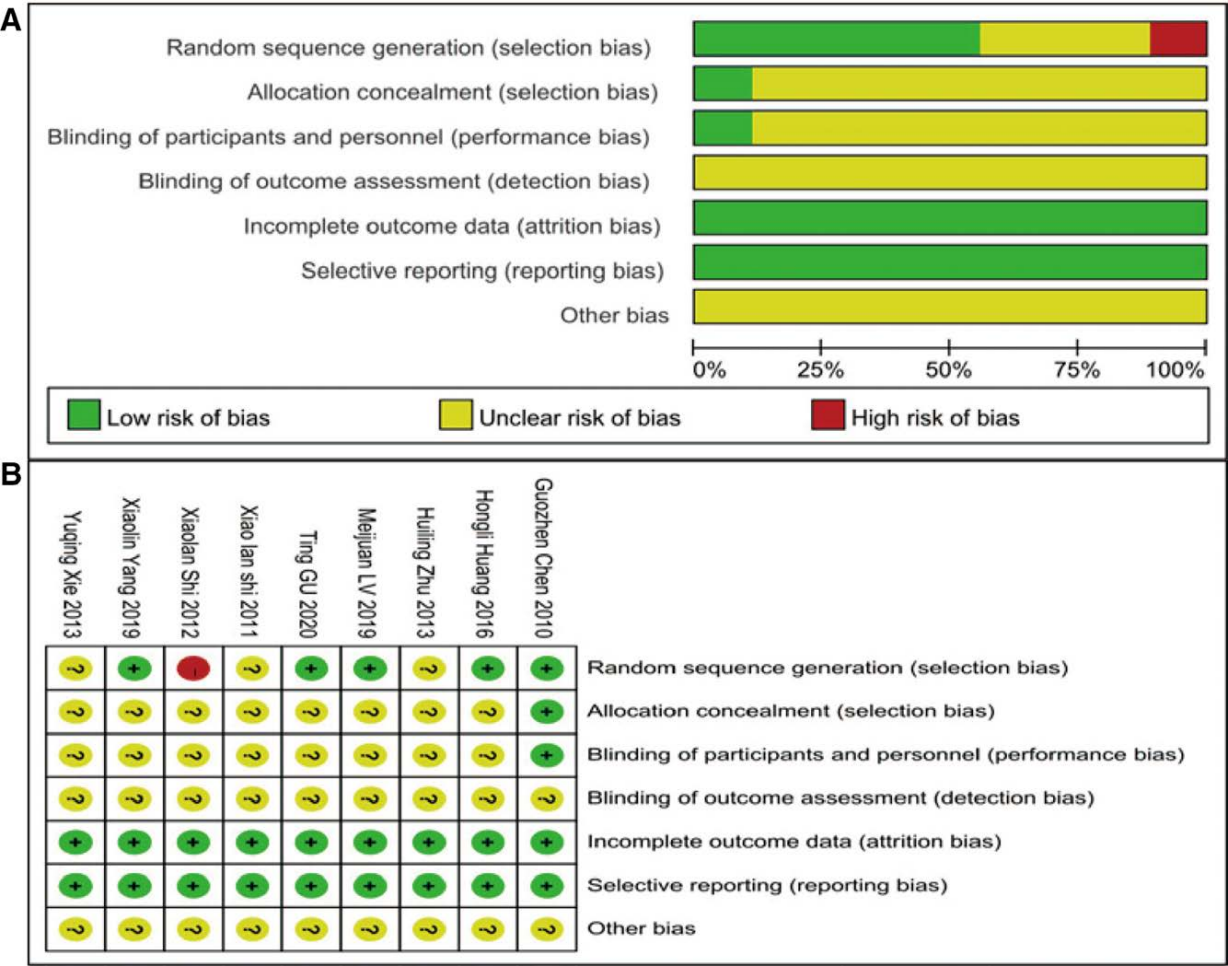
**Proportion of included research projects with risk of bias**. **(A**) Risk of bias summary, showing the review authors’ judgments about each risk of bias item for each included study. **(B**) Risk of bias graph, illustrating the proportion of studies with low, unclear, and high risk of bias across different categories, including random sequence generation, allocation concealment, blinding, and selective reporting.

Three studies^[17,23,25]^ adopted the random number table method and were rated as low risk. One study^[19]^ used the order of visits to be randomized and rated as high risk. 5 studies did not mention randomization methods and were rated as unclear. Two studies^[17,24]^ mentioned blinding and rated it as low risk. The rest of the research did not mention blinding, and the rating is unclear. One study^[20]^ used systematic randomization in concealment and was rated as low risk; the rest of the research did not mention this, and the rating is unclear. There are no missing data or selective reports in any of the studies. None of the studies mentioned other biases, which remains unclear (Fig. 4).

### 3.4. Meta result analysis

#### 3.4.1. Total clinical effective rate

A total of 7 studies, involving 478 patients, reported the overall clinical effectiveness rate. A subgroup analysis was performed due to the different interventions used in the control groups. The heterogeneity test indicated low statistical heterogeneity across studies (*P* = .51, *I*^2^ = 0%), and a fixed-effects model was applied for the meta-analysis.

**Figure 5.**
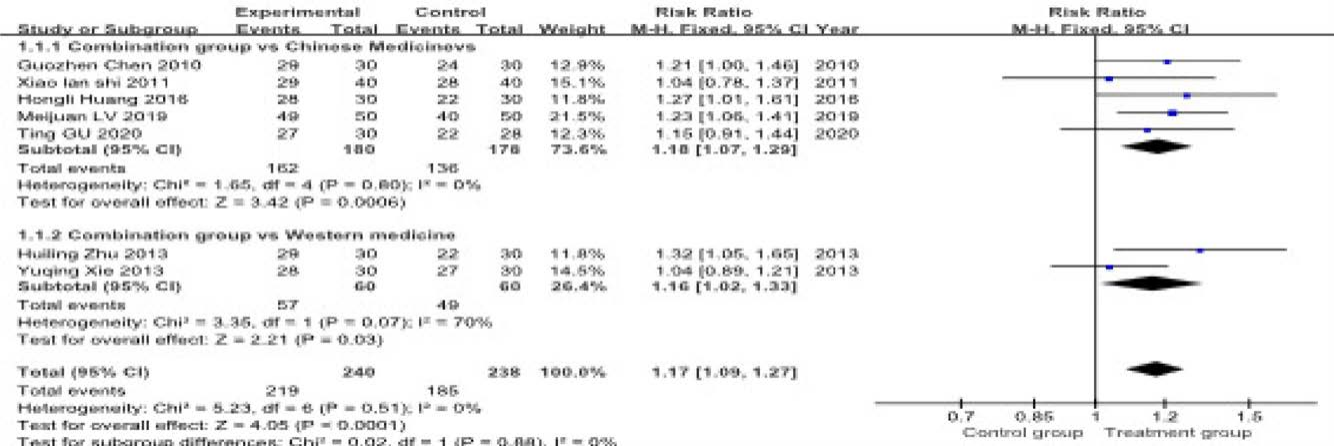
**Meta-analysis of clinical total effective rate.** This forest plot compares the clinical total effective rate between experimental and control groups in 2 subgroups: the combination group vs Chinese medicines and the combination group vs Western medicine. The risk ratio (RR) for each study is shown with 95% confidence intervals (CI). The overall RR for the Chinese medicine group is 1.18 (95% CI: 1.07, 1.29), and for the Western medicine group, it is 1.17 (95% CI: 1.09, 1.27). The heterogeneity for both subgroups is reported.

The pooled results demonstrated that acupuncture combined with Chinese herbal medicine significantly improved the total clinical effectiveness rate compared to monotherapies. The combined effect size was (RR = 1.17, 95% CI: 1.09–1.27). In subgroup analyses, the combination therapy was superior both to Chinese herbal medicine alone (RR = 1.18, 95% CI: 1.07–1.29) and to antidepressant drugs alone (RR = 1.16, 95% CI: 1.02–1.33) (Fig. 5).

#### 3.4.2. HAMD score

Seven articles, involving a total of 478 patients, reported the Hamilton Depression Rating Scale (HAMD) score. A subgroup analysis was performed due to differences in the control group interventions. The test for heterogeneity indicated minimal statistical heterogeneity across the studies (*P* = .51, *I*^2^ = 0%), therefore a fixed-effects model was utilized for the meta-analysis.

**Figure 6.**
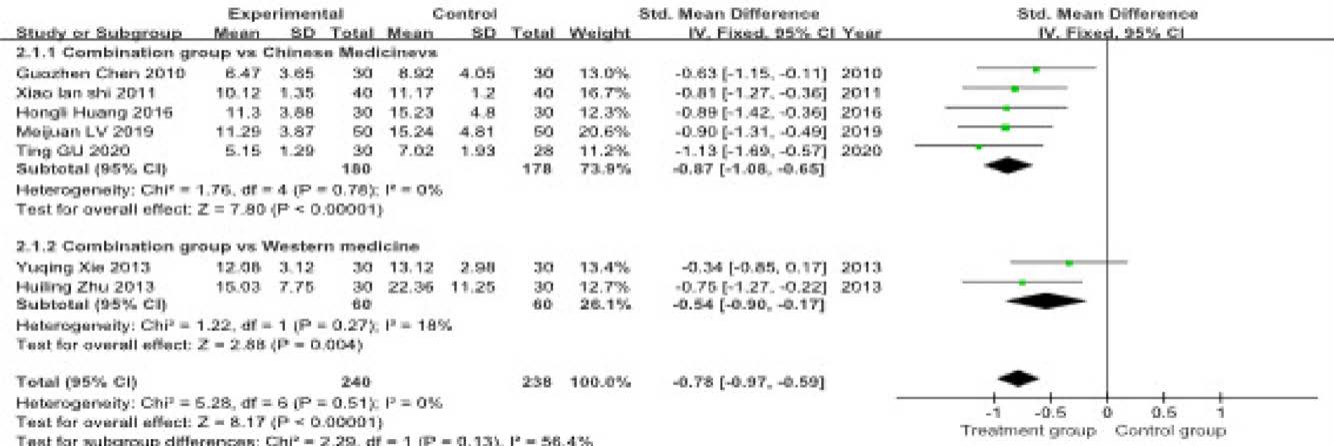
**Meta-analysis of HAMD score.** This forest plot compares the standardized mean difference (SMD) in HAMD scores between experimental and control groups in 2 subgroups: the combination group vs Chinese medicines and the combination group vs Western medicine. The overall SMD for the Chinese medicine group is −0.87 (95% CI: −1.08, −0.65) and for the Western medicine group is −0.54 (95% CI: −0.90, −0.17). The heterogeneity for both subgroups is reported. CI = confidence interval, HAMD = Hamilton depression scale.

The analysis revealed that the combination of acupuncture and Chinese herbal medicine was associated with a statistically significant reduction in HAMD scores compared to control interventions. The pooled effect size was (SMD = −0.78, 95% CI: −0.97–−0.59). Subgroup analyses indicated that the combined therapy was superior both to Chinese herbal medicine alone (SMD = −0.87, 95% CI: −1.08–−0.65) and to antidepressant drugs alone (SMD = −0.54, 95% CI: −0.90–−0.17) (Fig. 6).

#### 3.4.3. Kupperman index

Four studies, involving a total of 298 patients, reported the Kupperman Index score. The test for heterogeneity indicated substantial statistical heterogeneity across studies (*P* = .002, *I*^2^ = 80%), therefore a random-effects model was applied for the meta-analysis.

**Figure 7.**
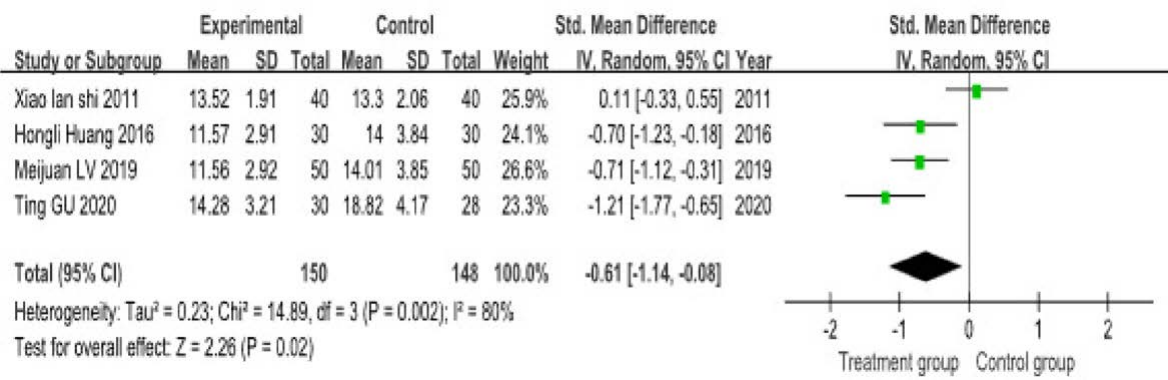
**Meta-analysis of the Kupperman index**. This forest plot shows the comparison of Kupperman index scores between experimental and control groups. The standardized mean difference (SMD) with 95% confidence intervals (CI) is presented for each study. The pooled analysis indicates a significant reduction in the Kupperman index in the experimental group compared with the control group (SMD = −0.61, 95% CI: −1.14–−0.08).

The pooled analysis demonstrated that acupuncture combined with Chinese herbal medicine significantly reduced the Kupperman Index score compared to the control groups (which received either Chinese herbal medicine or antidepressant drugs alone), with a combined effect size of (SMD = −0.61, 95% CI: −1.14–−0.08) (Fig. 7).

#### 3.4.4. Estradiol content

Four studies, involving a total of 260 patients, reported estradiol levels. A subgroup analysis was performed due to differences in control group interventions. The heterogeneity test indicated low statistical heterogeneity (*P* = .21, *I*^2^ = 33%), therefore a fixed-effects model was used for the meta-analysis.

**Figure 8.**
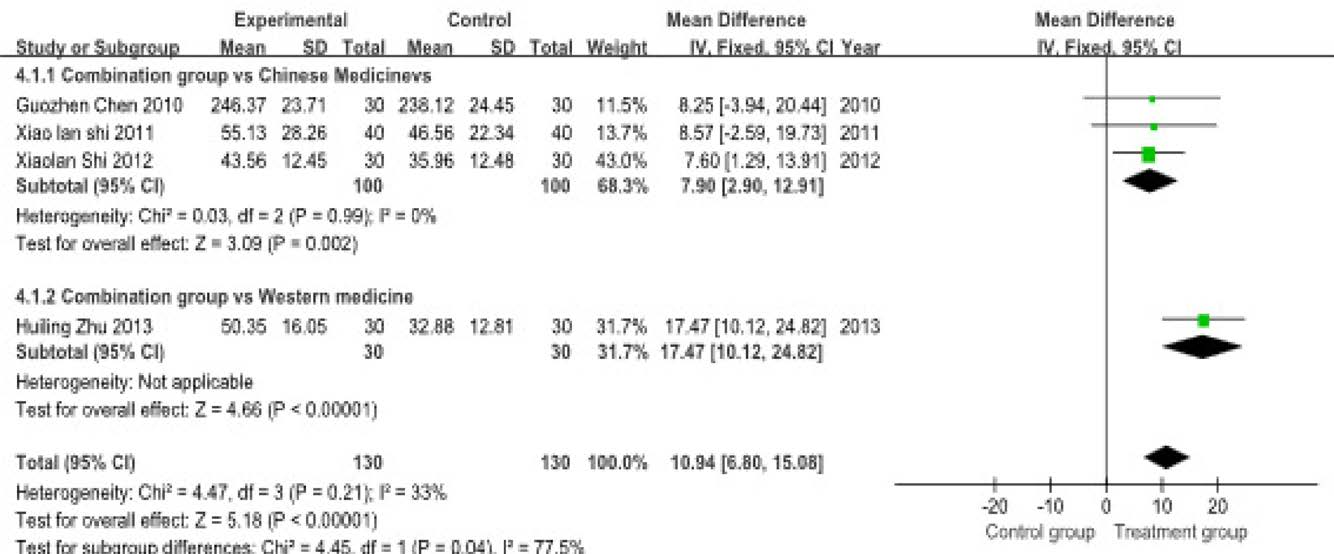
**Meta-analysis of estradiol content.** This forest plot compares the mean difference in estradiol content between experimental and control groups in 2 subgroups: the combination group vs Chinese medicines and the combination group vs Western medicine. The pooled mean difference for the Chinese medicine group is 7.90 (95% CI: 2.90, 12.91) and for the Western medicine group is 17.47 (95% CI: 10.12, 24.82). The heterogeneity for both subgroups is reported. CI = confidence interval.

The pooled results demonstrated that acupuncture combined with Chinese herbal medicine significantly increased estradiol levels compared to control therapies. The combined effect size was [MD = 10.94, 95% CI: 6.80 to 15.08]. In the reported subgroup analysis, the combination therapy was superior to Chinese herbal medicine used alone [MD = 7.90, 95% CI: 2.90–12.91] (Fig. 8).

#### 3.4.5. SDS scores

SDS scores were reported in 2 studies, involving a total of 121 patients. The heterogeneity test indicated substantial statistical heterogeneity across these studies (*P* = .03, *I*^2^ = 78%), therefore a random-effects model was applied for the meta-analysis.

**Figure 9.**
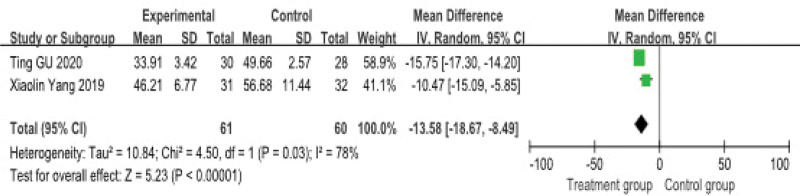
**Meta-analysis of SDS score**. This forest plot compares the mean difference in SDS scores between experimental and control groups. The pooled mean difference for the experimental group is −13.58 (95% CI: −18.67, −8.49). The heterogeneity across the studies is significant (*I*^2^ = 78%, *P* = .03), indicating variability in the results. CI = confidence interval.

The pooled analysis suggested that, compared to the control group, acupuncture combined with Chinese herbal medicine was associated with a significant reduction in SDS scores (MD = −13.58, 95% CI: −18.67–−8.49) (Fig. 9).

#### 3.4.6. Acupuncture interventions

Characteristics of acupuncture interventions summarizing the variability in techniques^[22]^: Needle depth (often described as “standard depth” or 10–30 mm), retention time (commonly 20–30 minutes), stimulation methods (manual twisting, electroacupuncture), frequency (typically once daily or every other day), and acupoint selection (common points like Baihui (GV20), Sanyinjiao (SP6), Hegu (LI4) were frequently used).

### 3.5. Adverse reactions

Huiling Zhu^[21]^’s control group had 5 cases of headache, nausea, and dizziness. Xiaolin Yang^[24]^ reported 1 case of adverse reaction occurred in the treatment group and 5 cases of adverse reaction events in the control group. No adverse events occurred in the remaining studies. The adverse reaction rate in the treatment group was 0.3%, while the adverse reaction rate in the control group was 3.3%. It can be seen that the safety of combined acupuncture and Chinese herbal medicine is better than that of TCM or antidepressant drugs.

### 3.6. Sensitivity analysis

The sensitivity analysis was carried out by eliminating single studies one by one. In the meta-analysis of the Kupperman index, after excluding Shi Xiaolan’s 2011^[18]^ research, *I*^2^ changed from 80% to 13%. After careful review of the literature and analysis, the cause of the heterogeneity may be differences in treatment duration or Kupperman’s different scoring rules.

### 3.7. Bias analysis

**Figure 10.**
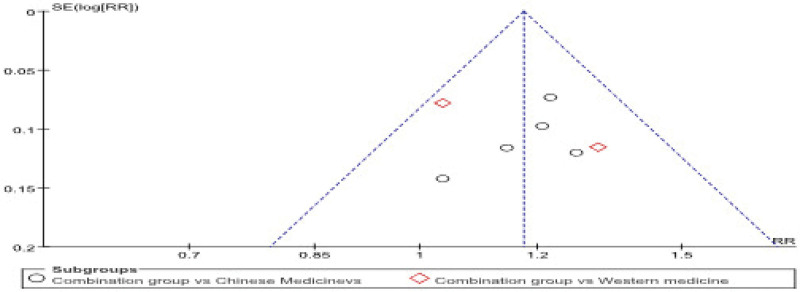
**Funnel chart**. This funnel plot assesses publication bias by comparing the standard error of the log-transformed risk ratio (SE(log[RR])) with the risk ratio (RR) for the experimental studies. The open circles represent studies comparing the combination group vs Chinese medicines, and the red diamonds represent studies comparing the combination group vs Western medicine. The symmetrical shape of the funnel suggests no significant publication bias.

The study uses a funnel plot analysis. Figure 10 shows that the funnel plot is symmetrical on both sides, suggesting that the risk of reporting bias is small.

## 4. Discussion

In Western medicine, the etiology and pathogenesis of perimenopausal depression remain incompletely understood. Currently, several theories regarding its pathogenesis are prevalent, including the estrogen withdrawal theory, domino theory, norepinephrine hypothesis, serotonin hypothesis, etc^[26]^ However, due to a lack of clear theoretical guidance, treatments such as antidepressant drugs and HRT suffer from drawbacks including slow onset, limited efficacy, and poor patient compliance. Additionally, commonly used medications like Prozac and Progynat may cause adverse reactions such as dizziness, nausea, and dependency.^[27]^

TCM categorizes perimenopausal depression into classifications such as “feminine dryness” and “lily disease,” with a long-standing history of understanding. TCM has developed a comprehensive theoretical framework covering etiology, pathogenesis, treatment principles, and methods. For instance, a 49-year-old woman in perimenopause may exhibit signs below: Tiangui is about to exhaust, and the 2 pulses of Chong and Ren are weak. The yin and yang of the kidney are imbalanced. If it is not adjusted in time, the yin and yang and blood of other organs will also be imbalanced, which will induce diseases. Insufficiency of kidney yin, kidney water can’t get up to the heart fire, heart fire up inflammation, disturbing the mind. Insufficient kidney yang, unable to instigate the rise of liver qi, loss of liver function, dysregulation of emotional dysfunction, emotional depression, reticence and other symptoms which potentially trigger additional diseases. According to Xiaojie Li’s synthesis of literature,^[28]^ perimenopausal depression primarily manifests as heart-kidney disharmony, kidney deficiency, and liver depression. Clinical treatment focuses on syndrome differentiation tailored to individual patient conditions. Studies by Li Sheng^[29]^ and Zhiliang Liu,^[30]^ employing meta-analysis and retrospective cohort studies demonstrate that acupuncture and Chinese herbal medicine outperform antidepressants or HRT in treating perimenopausal depression. While TCM’s efficacy in this regard has gained recognition in recent years, most treatments are predominantly monotherapy, with limited reports on combined therapies.

Chinese herbal medicine primarily regulates the visceral qi, blood, yin, and yang of the viscera to achieve therapeutic effects, albeit with a slow onset and occasional poor patient compliance.^[31]^ Acupuncture, on the other hand, balances meridian qi, blood, yin, and yang swiftly and effectively, although some patients experience needle fainting, which limits its applicability.^[32]^ Combining acupuncture with Chinese herbal medicine compensates for single treatment drawbacks and demonstrates the advantages of multi-target, multi-channel, and multi-level therapy.^[33]^ Based on the analysis in this article, the Bushen Jieyu Qingxin prescription combined with acupuncture at Sanyinjiao, Guanyuan, Sishencong, and Baihui acupoints yields optimal therapeutic outcomes. However, the specific treatment plan depends on clinical differentiation.

The comprehensive analysis of this study indicates that, compared to the Chinese herbal decoction alone or Western medicine control groups, the intervention group receiving acupuncture combined with Chinese herbal decoction demonstrates advantages in improving the primary clinical outcomes for patients with perimenopausal depression. Specifically, this is reflected in a higher overall clinical effectiveness rate, more significant reductions in Hamilton Depression Rating Scale (HAMD), Kupperman Index, and SDS scores, as well as an increase in estradiol (17β-estradiol) levels. Importantly, the combined therapy shows a favorable safety profile with a lower incidence of adverse events. These findings align with the current international exploratory trend in integrative medicine for managing comorbid emotional and reproductive endocrine disorders. Growing evidence suggests that acupuncture may exert antidepressant effects through multiple mechanisms, such as regulating HPA axis function, reducing neuroinflammation, and promoting neuroplasticity.^[34]^ Concurrently, specific Chinese herbal formulas may synergistically modulate neurotransmitter systems, estrogen receptor signaling pathways, and the overall internal homeostasis of the body via a “multi-component, multi-target” approach.^[35]^ The synergistic effects observed in this study provide clinical evidence supporting the notion that these 2 therapies have complementary or additive effects at the neuroendocrine regulatory level. This suggests their combined application may constitute a multimodal intervention strategy targeting the complex pathophysiological mechanisms of perimenopausal depression.^[36]^

It is noteworthy that perimenopausal mood disorders are closely associated with ovarian function decline and fluctuations in estrogen levels. The results of this study show that the combined treatment is superior to some control groups in elevating estradiol (17β-estradiol) levels, implying that its therapeutic effect may be related to the modulation of gonadal axis function to some extent. However, the exact mechanisms: whether it acts directly on the ovaries, provides feedback regulation to higher centers, or indirectly affects endocrine function by improving overall condition, still require elucidation through future basic research. Although the findings of this study are positive, caution is warranted when translating them into broad clinical practice guidelines. The main limitations include the overall methodological quality of the included studies, variations in specific intervention details (such as acupuncture manipulation techniques and herbal formulations), and restrictions related to study populations and follow-up durations.

We now explicitly discuss: Methodological quality: The risk of bias was high or unclear in several domains, particularly regarding blinding of participants and personnel. The nature of the interventions (acupuncture vs drug) makes effective blinding challenging, which may have influenced subjective outcome assessments like depression scores. Regarding publication bias: Although Egger’s test did not indicate significant publication bias for our primary outcomes, the test’s power is limited by the small number of studies. The potential for unpublished negative or neutral results remains a concern. Other added limitations include the aforementioned intervention heterogeneity and the short-term focus of most studies.

Upon comprehensive analysis of the included studies, several limitations were identified in the literature: First, the generalizability of our findings may be constrained by the geographical concentration of the included studies, which were predominantly conducted in China. Additionally, variations in treatment durations across trials could influence the stability and comparability of the outcomes. Further clinical heterogeneity arose from differences in acupuncture protocols, including point selection, needle manipulation, angle of insertion, and retention time, as well as in the specific compositions of the TCM decoctions administered. While this variability reflects real-world clinical practice, it complicates the derivation of a unified, standardized treatment protocol from our results. Finally, due to the limited number of available studies, we were unable to perform subgroup analyses based on specific pharmaceutical agents, which warrants further investigation in future research with larger sample sizes. The literature search for this study was conducted up to April 2021 and did not include clinical trials published thereafter. While this aligns with the predefined study protocol, it may result in the conclusions not fully reflecting the most recent advancements in the evidence base of this field.“ We consider the present study an important interim achievement and a valuable evidence benchmark. Regarding new evidence emerging after April 2021, our team has planned to incorporate it as the core focus of a subsequent independent study or an updated version of this systematic review. We intend to conduct in-depth analysis and comparisons of the newly added evidence to dynamically and continually enhance the understanding of this clinical issue.

## 5. Conclusion

The available evidence suggests that combining acupuncture with TCM decoction effectively treats patients with PDD. Meta-analysis results support this treatment modality as potentially optimal for managing PDD. Furthermore, acupuncture combined with TCM decoction appears safer and more effective in treating PDD compared to using TCM decoction alone or Western medicine. However, large-scale and high-quality clinical studies are necessary to further validate its efficacy.

These findings suggest that the integration of acupuncture with TCM decoction could be considered as a potentially effective and well-tolerated therapeutic strategy for PDD in clinical settings, particularly for patients seeking non-pharmacological options or experiencing side effects from conventional antidepressants. Future RCTs should prioritize rigorous methodology, including adequate sample sizes, explicit randomization and allocation concealment, and attempt blinded outcome assessment where feasible. Standardized or pragmatic yet replicable protocols for both acupuncture and TCM decoction are needed to improve comparability across studies. Investigations into the long-term efficacy and sustainability of treatment effects, as well as studies exploring potential mechanisms (e.g., effects on the HPA axis, neuroinflammation, or neuroplasticity), are warranted. Cost-effectiveness analyses comparing this combined approach with standard care would be valuable for healthcare decision-making.

## Acknowledgments

The authors declare that there was no funding and no conflict of interest for this study.

## Author contributions

**Data curation:** Wenya Huang, Le Zhang, Zi Wang.

**Resources:** Wenya Huang, Zhan Gao.

**Supervision:** Wenya Huang, Zhan Gao.

**Formal analysis:** Le Zhang, Yue Dong.

**Software:** Le Zhang, Zhonghao Fang, Zhan Gao.

**Funding acquisition:** Zhonghao Fang, Kuok Tong Lei.

**Methodology:** Zhonghao Fang, Yue Dong, Zhan Gao.

**Project administration:** Zhonghao Fang, Zi Wang.

**Investigation:** Yue Dong.

**Conceptualization:** Zhan Gao.

**Visualization:** Zhan Gao.

**Writing – original draft:** Wenya Huang, Le Zhang, Zhan Gao.

**Writing – review & editing:** Wenya Huang, Le Zhang, Zhan Gao, Kuok Tong Lei.

## Supplementary Material
