# Supplementary material for: Treatment of perimenopausal depressive disorder with acupuncture combined with traditional Chinese medicine decoction: A systematic review and meta-analysis
Source: Medicine (Baltimore). 2026 Apr 24;105(17):e48396. doi: 10.1097/MD.0000000000048396 (PMC13124357; doi:10.1097/MD.0000000000048396)
Supplement: Supplementary file 1 [file medi-105-e48396-s001.pdf]

# Supplementary Methods

## PubMed

1. ("Climacteric" [MeSH Terms] OR "Perimenopause" OR "Depression" OR "Depressive Symptoms" OR "Emotional Depression")
2. AND ("Chinese Herbal Medicine" OR "Traditional Chinese Medicine" OR "Acupuncture" OR "Electro-Stimulation" OR "Scalp Acupuncture" OR "Needle")
3. Filters: RCTs, published up to April 2021.

## Web of Science

1. TS=("Climacteric" OR "Perimenopause" OR "Depression" OR "Depressive Symptoms" OR "Emotional Depression")
2. AND TS=("Chinese Herbal Medicine" OR "Traditional Chinese Medicine" OR "Acupuncture" OR "Electro-Stimulation" OR "Scalp Acupuncture" OR "Needle")
3. Document Types: RCTs, Date: Until April 2021.

## Embase

1. ('Climacteric'/exp OR 'Perimenopause' OR 'Depression'/exp OR 'Depressive Symptoms' OR 'Emotional Depression')
2. AND ('Chinese Herbal Medicine'/exp OR 'Traditional Chinese Medicine'/exp OR 'Acupuncture'/exp OR 'Electro-Stimulation' OR 'Scalp Acupuncture' OR 'Needle')
3. Filters: RCTs, published until April 2021.

## The Cochrane Library

1. ("Climacteric" OR "Perimenopause" OR "Depression" OR "Depressive Symptoms" OR "Emotional Depression")
2. AND ("Chinese Herbal Medicine" OR "Traditional Chinese Medicine" OR "Acupuncture" OR "Electro-Stimulation" OR "Scalp Acupuncture" OR "Needle")
3. Filters: RCTs, published up to April 2021.

## Sinomed

1. ("围绝经期" OR "更年期" OR "抑郁" OR "抑郁症状" OR "情绪抑郁")
2. AND ("中药" OR "中医药" OR "针刺" OR "电针" OR "头皮针" OR "针灸")
3. 检索类型：随机对照试验（RCT），检索时间截至 2021 年 4 月。

**CNKI, Wanfang, VIP**

1. ("围绝经期" OR "更年期" OR "抑郁" OR "抑郁症状" OR "情绪抑郁")
2. AND ("中药" OR "中医药" OR "针刺" OR "电针" OR "头皮针" OR "针灸")
3. 检索类型：随机对照试验（RCT），检索时间截至 2021 年 4 月。
